# Commentary: how person-centred is pharmaceutical care?

**DOI:** 10.1007/s11096-021-01332-0

**Published:** 2021-09-25

**Authors:** Hanna Gyllensten, Joanne M. Fuller, Malin Johansson Östbring

**Affiliations:** 1 Institute of Health and Care Sciences, Sahlgrenska Academy, University of Gothenburg, Box 457, 405 30 Gothenburg, Sweden; 2 Centre for Person-Centred Care (GPCC), University of Gothenburg, Box 457, 405 30 Gothenburg, Sweden; 3 eHealth Institute, Department of Medicine and Optometry, Linnaeus University, Kalmar, Sweden; 4 Pharmaceutical Department, Region Kalmar County, Kalmar, Sweden

**Keywords:** Clinical pharmacy, Patient care management, Patient-centred care, Pharmaceutical services, Pharmaceutical care, Person-centred care

## Abstract

Health systems in many countries are currently undergoing an evolution towards more person-centred care. However, an overview of the literature shows that there is little or no guidance available on how to apply person-centred care to pharmaceutical care and clinical pharmacy practices. In this paper we apply a model for person-centred care created by a national multidisciplinary research centre in Gothenburg, Sweden, to the clinical work tasks of outpatient and inpatient pharmacists and describe how pharmaceutical care can become more person-centred.

## Impacts on practice

- As part of the health system, pharmacists and other healthcare professionals providing pharmaceutical care are well-placed to adopt a person-centred ethic.
- The different roles and responsibilities of clinical pharmacists and community pharmacists will affect the further development of person-centred care, in initiating, working, and safeguarding the partnership with the patient.
- One of the challenges for a health system evolving towards more person-centred care will be the inclusion of pharmacists in oral and written communication between and within care providers.

## Commentary

### Aim

The aim of this commentary is to provide insight on how the current evolution towards person-centred care in many health systems could impact on and be incorporated into contemporary pharmaceutical care practices.

### What is person-centred care?

Health systems are currently evolving towards more person-centred care (PCC), as indicated by recently published international standards for patient participation in PCC [1]. Pharmacists have different roles in the health system, from medication and medical device supply to providing pharmacotherapy support for patients. Thus, we believe pharmacists in their supportive role to patients as persons regardless of their disease status, should provide person-centred pharmaceutical care.

The current consensus, among advocates for using the terminology of patient-centred and person-centred care alike, is that it is beneficial for patients to be more involved in their healthcare [2]. The concept of person-centredness has been suggested to have evolved at a different pace in different aspects of medical care since the 1960s, with the term person-centredness being used more often in nursing [2]. Person-centredness relates to a meaningful life and differentiates from patient-centred care that puts emphasis on a functional life [3]. In many instances their use appears interchangeable [2], but some authors favour the term person-centred over patient-centred. The later may indicate a more narrow interaction between professional and patient, limiting patients to their role as patient, and disregard the person within their context [2].

This discussion applies to countries in which PCC is becoming mandated on some levels and is potentially more applicable to countries where healthcare is largely publicly funded. It has been reported that it is more common in literature from European countries using the Beveridge model for funding their health system to include the concept of PCC [4]. Our experiences derive from the Swedish context. In Sweden, healthcare is predominately financed through taxes, and is organised in 21 regions of which the majority have an ongoing discussion or decisions about introducing PCC. Moreover, the Swedish health and social care sector has embraced a model for PCC developed by the University of Gothenburg Centre for Person-centred Care (GPCC), which is a research centre for PCC in chronic diseases. This model, sometimes referred to as “the Gothenburg model” [5], strategically focuses on developing sustainable healthcare for all and is based on the underpinning ethic that focuses on the person and their capabilities. The model entails three core concepts relevant to clinical practice: the patient narrative, partnership building between the patient and healthcare professional (HCP), and safeguarding of the partnership through documentation [5].

### Are pharmacists that practice pharmaceutical care ready to provide person-centred care?

When the professional role of pharmacists changed over the last century, taking on a responsibility for patient care [6], pharmaceutical care was defined in a way that connects provision of pharmacotherapy to the purpose of relevant outcomes for patients. The pharmaceutical care approach specifically points to the cooperation between patient, pharmacist, and other professionals in designing, implementing, and monitoring the therapeutic plan and outcomes, and that the pharmacist is directly responsible to the patient. A central concept of care is caring, and a one-to-one relationship between caregiver and patient [7, 8]. Already in the early 2000s, the need to explicitly define caring behaviours relevant to pharmaceutical care was identified [7].

Collaborative aspects of pharmaceutical care are emphasised in standards, mainly focusing on the professional team but acknowledging also the importance of the relationship with the patient [8]. In the American Pharmacist Association’s more recent definition, pharmaceutical care is described as patient-centred, where pharmacists “work in concert with the patient” and other professionals [9]**.** There are, however, several documents using the person-centred terminology, but with limited information on the conceptual or practical changes necessary for this development. For example, in strategic documents from the International Pharmaceutical Federation (FIP), pharmaceutical care is very much part of the pharmacist’s role, and one of their strategic outcomes is to “support and empower pharmacists to provide high quality person-centred pharmaceutical care to improve health outcomes for individuals and populations alike”. A recent paper by Uzman et al. [10], states that a shift has occurred, from a traditional medicine-centred approach in pharmacy, to an advanced person-centred approach. However, further clarification is limited to that “pharmacists working in the community play a crucial role in providing the human touch and bringing patient-centricity to the forefront”. Most documents about pharmaceutical care and the pharmacist’s new role focus on resolving pharmacotherapy problems and the outcome of safer medication use; as such, they stem from the aim to increase productivity and efficient use of resources rather than the underpinning ethic of person-centredness.

Pharmacists are trained as medication experts where their role involves ensuring patients’ adherence to prescribed medicines. However generally, this role has not included the patient as an active partner in their care. A degree in pharmacy stems from a positivist discipline where scientific evidence is seen as the guiding principle to be adhered to, or the one and only correct truth [11]. Hence, pharmacists, in their role as ‘keepers’ of ‘good medication-taking’, work in a predominantly positivist manner, and base their clinical practice on evidence-based treatment in the form of guidelines. Whilst correct from a biomedical aspect, this approach remains systems-based where the patient is typically seen as a passive recipient of their treatment. Yet patients live in a constructivist world as opposed to a positivist world. A constructivist world includes the complex intersections of social life that influence a patient’s actions (e.g., medication-taking). Patients’ actions or behaviours are a result of their decision-making that is in turn directed by how they experience medications [12] and their relationship with health professionals [13].

A person-centred approach recognises the patient’s experiences in the care context and entails involving the patient as an active partner, or as exemplified by the Gothenburg model: initiating a partnership between the patient and the HCP. To initiate the partnership, the pharmacist must acknowledge the patient’s narrative. For example, in terms of medication usage, acknowledging the patient’s social situation and their experiences with medications, and if they have existing strategies around health and medications. The next key step is to work this already initiated partnership and support pharmacotherapy that best fits the patient based on both their clinical status and social situation as identified through the patient narrative. Here it is appropriate to work towards a co-created reciprocal agreement [14]. Putting the person and the partnership first can increase the use of effective, evidence-based medications [15].

Parallel with professional organisations’ focus on pharmaceutical care, the focus on patient adherence [16] has facilitated countless adherence interventions. Relying on complex behaviour theory, and on the concept of concordance and shared decision-making, the involvement of patients as active partners has often been emphasised [17–19]. Although a biomedical approach dominates these interventions, aspects of person-centredness are more pronounced in these practices when compared with clinical pharmacy services that focus mainly on medication reviews. In areas other than pharmacy where PCC is more pronounced, this evolution also stemmed from gradual changes in practice with initial aspects of person-centredness prior to the inclusion of more explicit partnership and co-creation [2, 5, 20]. To be person-centred, a medication review should start with a dialogue in which the pharmacist elicits the patient narrative through enquiring about their medication experience, expectations, and preferences, and seek a common understanding of the goals and conditions. Assuming some of the previous studies have included such narratives even when not clearly stated, there is still seldom a structure for co-creating a medication plan and less so, a structure for following-up this plan. Nonetheless, the follow-up has gained increased attention over the last decade. In Sweden, the Medbridge trial [21] investigated the effects of hospital-based comprehensive medication reviews where one arm included post-discharge follow-ups, but found no effect on patients’ unplanned hospital visits within 12 months when compared to a control group. In contrast, a Danish study of a similar intervention explicitly used the patient-centred technique of motivational interviewing in their follow-up; they found that readmissions within 30 and 180 days were decreased among patients who received medication reviews and follow-up [22]. Although there are several differences between the interventions tested in these two trials, we suggest that the difference in how the follow-up was performed may explain the different results. In addition to motivational interviewing, the GROW coaching model has been suggested useful for supporting person-centred conversations and to encourage patients in becoming active participants in their care [23].

For pharmacists, however, expected by their employer to provide PCC, there is a lack of clear guidance on how to practice PCC in pharmacy. In the UK, the National Health Service’s (NHS) policy documents state the need to move to PCC as a concept within medicines optimisation but concrete guidelines differentiating it from patient-centred practices remain absent, at least in the documentation available outside of that organisation.

### Towards a more person-centred pharmacy practice

**Fig. 1:**
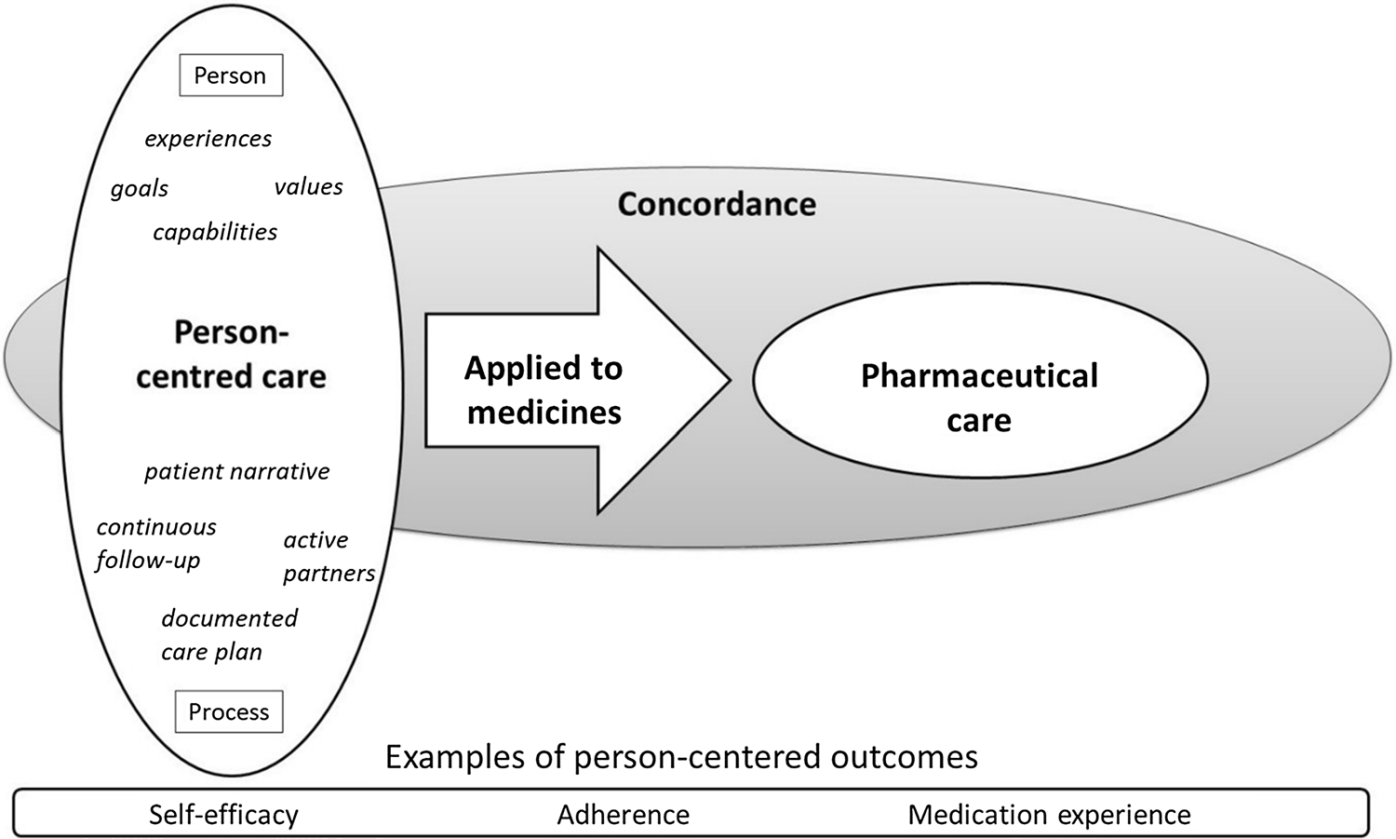
Pharmaceutical care as described by Cipolle et al., related to concordance and GPCC (from Johansson Östbring 2021 [24], with permission)

Many research studies on PCC have had a multi-professional approach, including all aspects of health, yet there are also studies applying PCC in the tasks of a specific staff category [20]. When applied to medications and pharmaceutical care, PCC will ultimately be about the patients’ medication experiences and how these can be aligned with their values in life and their available treatments [24]. In Fig. 1, we propose that when an underpinning ethic of PCC is applied to medications, it closely resembles the concept of pharmaceutical care as described by Cipolle et al*.* [25]. Hence, pharmaceutical care as described by Cipolle et al*.*[25] is a practical guide on how to practice person-centred medicines optimisation. The three cornerstones of person-centredness as described by Ekman and colleagues in the Gothenburg model can be used to operationalise this process; through *initiating, working, and safeguarding* the partnership. In applying the Gothenburg model [5] to pharmacotherapy, the narrative of the patients’ medication experiences is aligned to not only the patient’s experiences and capabilities but also with their values in life and available treatments when optimising pharmacotherapy. Along these lines, the Integrated Medication Management-initiative has introduced the concepts of patient experiences, partnership, and agreement [26]. Also, both PCC and pharmaceutical care use the process of concordance and is founded in evidence-based medicine. Below we provide practical guidelines on how these cornerstones can be applied to pharmaceutical care; in which the patient’s medication experience is defined as the starting point, and in line with the Gothenburg model, we emphasise documentation of the agreed plan and continued follow-up as a prerequisite for practicing pharmaceutical care.

In inpatient care settings, pharmacists are part of a larger team providing care, hence, the pharmacist is often not the first HCP to interact with an admitted patient. Ideally this patient has already received PCC, using the GPCC cornerstones of *initiating* and working the partnership and documentation. This model has been used in many different inpatient populations and settings [20], with beneficial outcomes [27]. With these steps in place, the pharmacist is provided with an in-progress co-created health plan. For clinical pharmacists within the care team, this entails approaching the patient and being able to refer to an existing health plan, where further agreements made through (*working* the) partnership can be incorporated in that plan (*safeguarding*). On the other hand, for pharmacists involved as consultants in medication reviews, without direct patient contact, PCC is provided by other actors. Thus, in this case, PPC only changes which documentation is provided to the pharmacist, to potentially include a health plan with the patient’s goals and preferences. This process makes the documentation and follow up of health plans formalised, thus differing from consultation guidelines for clinical pharmacists in the UK’s NHS, which lack reference to any previously written health plan [23].

In some outpatient care settings, the pharmacist is a more independent actor with ample possibility to *initiate* and *work* the partnership and set up the health plan (*safeguarding the partnership*) [24], thus including all three cornerstones in the Gothenburg model. Outpatient care, including preventive care and health promotion, appears to be particularly suitable for PCC, transforming the role of HCPs and strengthening the patients’ position [28, 29]. If using a holistic perspective and focus on patients’ individual needs and expectations, pharmacists established within the healthcare team can provide pharmaceutical care that is person-centred, as the partnership and co-created health plan is also documented in the patient’s medical record [30].

Although community pharmacy is well-placed in “Getting the Drug Therapy right for Each patient” through person-centredness, the community pharmacy sector must be governed to strengthen its positioning in the healthcare chain [31]. Community pharmacists are registered healthcare practitioners that are uniquely accessible (no appointment is needed), enabling more spontaneous discussion on patients’ health issues and any negative medication experiences. Through *initiating*, *working*, and *safeguarding* the partnership between community pharmacists and patients, the identified patient narratives can be incorporated into a health plan that already exists or form a new health plan. This plan can thereafter guide patients at home or in discussion with clinicians in finding optimal treatment through co-creation. We are yet to find examples in the literature of community pharmacists working in this manner with the expressed aim to provide person-centred care.

However, there are some obvious obstacles in the path towards more PCC, such as professional attitudes and hierarchical structures [5], which applies to all HCPs in health systems evolving towards care that is more person-centred. Moreover, pharmacists often work in separate organisations to other HCPs, thus increasing the need for transfer of documented health plans within and between actors. One solution is that the health plan resides with the patient, but it is still likely that PCC will result in further demand for additional (electronic) communication between care providers, particularly between prescribers and pharmacies, through high security systems that ensure confidentiality of data. A similar challenge remains in insufficient knowledge available on how different members of professional teams will collaborate to provide their end of the partnership; pharmacists are not expected to be the sole carer of a patient, and thus are always part of a larger team with the patient and other HCPs. Whilst PCC has often resulted in beneficial [5, 27, 32], even cost-effective [33–36], results in randomised controlled trials, there are costs associated with implementation, including increased time spent in initiating the partnership. Thus, many questions remain as to how pharmaceutical care can become more person-centred, such as financial aspects and the acceptance of the changing role among pharmacists, other HCPs, and not least, amongst patients. Hence, further research conducted together with users of our pharmaceutical care is warranted.

## Conclusions

In this commentary, we advocate a change towards a more person-centred approach to pharmaceutical care and provide some suggestions on how this can change pharmacy practices across different settings. Pharmaceutical care documents do not traditionally include the cornerstones of PCC as described by the Gothenburg model, being the narrative, the partnership, and the co-created health plan. Thus, we believe that considerable work remains to enable pharmacists to practice PCC. If pharmacists and pharmacies claim to be part of healthcare, such a process is necessary as health systems in Europe further develop towards PCC.
